# An All-Textile Non-muscular Biomimetic Actuator Based on Electrohydrodynamic Swelling

**DOI:** 10.3389/fbioe.2020.00408

**Published:** 2020-05-19

**Authors:** Ilmar Uduste, Friedrich Kaasik, Urmas Johanson, Alvo Aabloo, Indrek Must

**Affiliations:** Intelligent Materials and Systems Lab, Institute of Technology, University of Tartu, Tartu, Estonia

**Keywords:** electro-active textiles, mechano-active textiles, biomimetic, wearable robots, ionic actuators, electrohydrodynamics, swelling, carbon

## Abstract

Mass transfer from one part of an organism to another constitutes a fundamental non-muscular movement strategy in living organisms, in particular in plants. The demonstrable simplicity and safety make non-muscular actuators especially attractive for distributed configurations such as in wearable robotic applications on a textile platform. However, practical arrangements for integrating actuators as inherent parts of textiles is an ongoing challenge. Here we demonstrate an electrohydrodynamic ionic actuator that combines two textiles of natural origin. The first textile — viscose-rayon-derived activated carbon cloth — consists of high-surface-area monolithic fibers that provide electrical and mechanical integrity, whereas the other textile – silk – contributes to mechanical integrity in the lateral direction while preventing the conductive textiles from contacting. By injecting an electronic charge into the activated carbon cloth electrodes, the migration of the electrolyte ions is initiated in the porous network in-between the electrodes, causing non-uniform swelling and eventually bending of the laminate. The three-layer laminate composed of integral textile fibers demonstrated a ∼0.8% strain difference. Electrical control over a fluid movement in a textile platform provides a scalable method for functional textiles not limited to actuation.

## Introduction

Wearable robotic appliances – i.e., devices to be worn on human bodies – promise to revolutionize personal healthcare, facilitate post-injury recovery, compensate for impaired bodily functions, and assist workers subjected to repetitive tasks in a factory setting, etc (Cianchetti et al., 2018)., Seamless integration of robots on our bodies also innovates our perception of our clothes, technologically similar since the industrial revolution in textile fabrication. On the contrary, the ongoing development of wearable robotics primarily stems from conventional mechatronics: the engineers are engaged in exploratory search of flexible functional analogs for the traditional building of robotics such as engaging rotary motors, gearwheels, levers, pistons, etc. The primary challenge in making robotics wearable is achieving the necessary mechanical, electrical, and chemical compliance to human bodies, showing a wide stiffness range (Majidi, 2014). The well-developed textile technology promises a platform for robots that have mechanical characteristics similar to human skin, yet packed with active elements (Han and Ahn, 2017; Maziz et al., 2017; Lee et al., 2020).

Stemming from the origins in the use of fluidics in mechatronics, the state-of-the-art in wearable robotics includes hollow structures with deformable boundaries (Polygerinos et al., 2017; Cacucciolo et al., 2019). Specific actuation characteristics are defined by the asymmetric placement of thin layers of more rigid materials or homogenous textile layers or fibers arranged in a more application-specific pattern (Sholl et al., 2020). Inflatable elastomer-fiber composite structures do mimic the muscular hydrostats common in animals, however, controlled supply of fluids to an effector from a remote reservoir and pump constitutes one major bottleneck for actuator performance in the textile setting. Besides biomimetic hydrostats that rely on exertion of hydraulic pressure on relatively inextensible boundaries, liquid movement can induce motion via other biomimetic mechanisms such as swelling. Actuators based on swelling of hygroscopic materials exposed to humidity gradients (Shin et al., 2018; Hu et al., 2019) provide straightforward and biomimetic design and utilize abundant biomaterials as well as humidity as the energy source, yet suffer in control challenges. A practical, micro-scale liquid-pumping solution is needed. Localized fluidic actuation by an electric input (Zhou et al., 2016; Hossan et al., 2018; Cacucciolo et al., 2019) is an attractive solution: by dislocating a hydraulic medium within a porous material *in situ* using an electrical input, the same part of material acts as a pump as well as an effector, diminishing the boundary in-between actuators and effectors. For localized pumping, electroosmotic separation of anions and cations of different size and mobility is an attractive solution. In essence, the actuator appears as being composed of thousands of miniature hydraulic pumps, each with a dimension defined by the size of a single pathway through the porous network, engaged in parallel. Leveraging to the examples in biological cells and tissues, cross-membrane ion- and water-pumping proteins are abundant for efficient, widely distributed fluidic action. Having highly localized fluid management, low-pressure dislocation of the liquid has the potential of inducing reversible swelling, as in hygromorphic bilayers. Indeed, the low pressure needed for inducing swelling, combined with few-micrometer-scale control of liquid movement direction by ion-pumping approach, can be more favorable for localized actuation in wearables, as leveraged to hydrostat-inspired solutions.

For a practical exclamation of directional electrohydrodynamic effects in a submillimeter-thickness structure with open porosity, electrodes need to be applied to its boundaries for a “source” and “sink” of ions. Useful approaches include maintaining ion current by electrochemical reactions at reactive electrodes. As leveraged to a more trivial hydrolysis-based approach, non-gassing reactive electrodes made of silver (Shin et al., 2011), as well as redox-active polymers (Erlandsson and Robinson, 2011), are potentially more practical. Nevertheless, capacitive, non-reactive electrodes promise systems of highest performance retention. Indeed, capacitive actuators with high-specific-surface-area (hi-SSA) activated carbon powder electrodes, based on electrolyte separation between the electrodes, have demonstrated excellent performance retention in cycling (Punning et al., 2014; Kaasik et al., 2017).

Nevertheless, as leveraged to polymeric composites with micrometer-dimension activated carbon particles, monolithic electrodes are favorable due to lower dependence on interparticle conduction. As even ultralong carbon nanotubes span only millimeters in their length, activated carbon cloth (ACC) represents a unique exception, demonstrating an uninterrupted hi-SSA conductive carbon network in length scale approaching to meters, covering the desired wearables’ range. A monolithic carbon electrode suggests superior electronic conductivity of the electrode, as electrons can travel via a carbon monolith from a power supply to any point on its surface, without interparticle electron transmission, to compensate the ionic charge at the electric double-layer. Moreover, as commercial hi-SSA ACC is typically synthesized from biomaterial (i.e., viscose) and considered safe for even wound dressing, ACC is an attractive robotic material.

Hi-SSA ACC electrodes, demonstrating flexibility similar to its precursor textile, have been previously engaged in an ionic (osmotic) actuator (Must et al., 2019), yet in a specific configuration where the electrodes were fixed to a static shape, where their flexibility did not avail. This research explores the possibility of constructing an electrohydrodynamic fluidic actuator based on monolithic flexible hi-SSA ACC textile as the electrode. In our previous work (Kaasik et al., 2017), we have approached the wearables’ field by building an actuator on a silk or glass fiber textile that did not compromise its tensile strength as a member of a fabric. The electrodes were sprayed on the membrane reinforced with an inert textile: although spray-application is industrially relevant and scalable, the electrodes did not form a monolithic electronically conductive carbon structure. This work proceeds by constructing an electroactive actuating laminate that combines high-strength inert textiles with hi-SSA conductive textile electrodes, thus best responding to the needs of the wearables’ technology.

## The Concept for Mechano-Active Textiles

### Materials of Biological Origin Used in an Electrically Active Biomimetic Laminate

The cues from the hierarchical and fibrous architecture of the mechano-active units in the plant and animal kingdoms are essential in rendering fibrous-but-static textiles as mechanically responsive. Fibrous chains transmit mechanical force in tendons, and elongated neuron cells carry information: consequently, the use of the fibrous nature of textiles is fundamental for biomimetic actuation.

**FIGURE 1:**
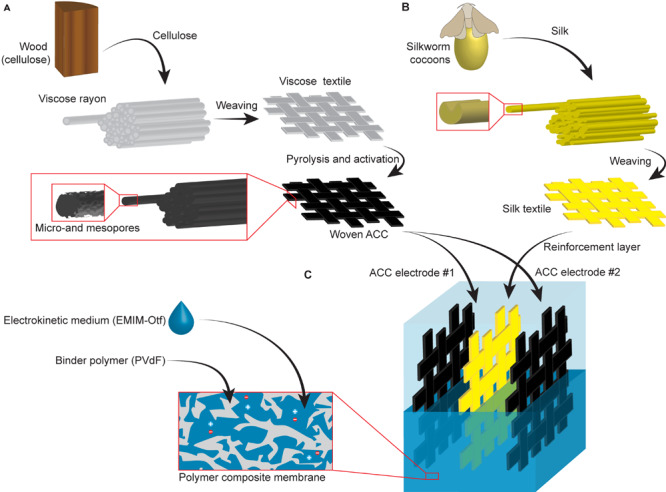
All-textile laminate. **(A)** The steps for producing viscose-rayon-derived woven activated carbon cloth (ACC). **(B)** The origin of the woven silk textile. **(C)** Structure of an ACC-silk-ACC laminate consolidated using a porous polymeric binder.

We designed a textile-based actuating laminate that engages two fibrous textiles of natural origin. First, viscose-rayon-derived ACC, with fabrication steps summarized in Figure 1A, acted as hi-SSA electrodes; the monolithic nature of its fibers also provided electronic signal integrity within the electrodes. Silk fabric (Figure 1B) provided tensile strength and mechanical unity.

An open-pore polymeric network merged a stack of three textile layers – a silk layer surrounded by two ACCs – into a laminate, as shown in Figure 1C. The porous polymeric network that formed during the fabrication hosted a liquid-state electrohydrodynamic medium that was free to move between different parts of the laminate.

### The Suggested Actuation Principle of the Laminate

**FIGURE 2:**
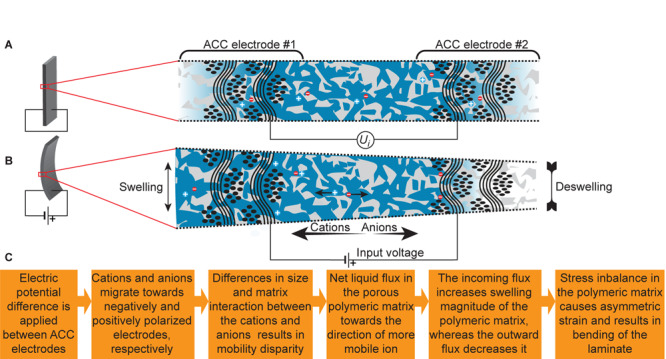
Working principle of the electrohydrodynamic actuator. **(A)** Initial state, showing the uniform distribution of electrolyte in the porous polymeric network. **(B)** Activated state, showing the non-uniform distribution of the electrolyte as a result of charge injection to the ACC electrodes. **(C)** The recalled sequence of the physical processes in an electrohydrodynamic actuator to convert the injected electronic charge into laminate bending.

Initially (depicted in Figure 2A), the electric double-layers on the surface of each ACC electrode contain a finite amount of charge, evidenced by finite electrode potentials. In a laminate with an ionic medium contained within the porous polymeric network, a finite voltage, *U*_*i*_, can be registered between two ACC electrodes. By applying a potential difference other than *U*_*i*_ in-between the electrodes, the cations and anions in the electrolytic medium start migrating toward the ACC electrodes to compensate their charge. As the cations and anions in the electrolyte differ in effective size and undergo specific interaction with the polymeric matrix and solvents, the cations and anions display asymmetric mobility. The more mobile ion pushes ahead and draws along several ions acting as a solvent, eventuating in a net flux of liquid in its migration direction. The resulting imbalance of liquid volume at the vicinities of oppositely polarized electrodes results in asymmetric swelling extent within the laminate; the resulting local strain difference is also expressed as bending of the laminate, as depicted in Figure 2B. The proposed steps in the actuation process are recalled in Figure 2C.

Plant-inspired actuation mechanisms based on physicochemical processes like osmosis and asymmetric swelling are sometimes referred to as nonmuscular (Must et al., 2019), as leveraged to the use of contractile proteins in animal muscles (Dumais and Forterre, 2012). The non-muscular actuators with the working principle given in Figure 2 are suggested for engineering applications where a high level of chemical, physical, and electrical safety is required, and performance characteristics comparable to plants are acceptable.

## Experimental

### Casting Solution for Polymeric Composite With an Electrohydrodynamic Medium

We dissolved 2 g of poly(vinylidene fluoride) (PVdF, M_w_ ∼534,000, Merck), 2 g of 1-ethyl-3-methylimidazolium trifluoromethanesulfonate (EMIM-Otf, 99.5%, Solvionic), and 4 ml of propylene carbonate (PC, Alfa Aesar) in 18 ml of N, N-dimethylacetamide (DMAc, Merck) by heating 24 h at 70°C under constant stirring.

The casting of the solution and evaporation of the solvents resulted in the formation of a polymeric open-pore network with its pores already (partially) filled with a solution of EMIM-Otf ionic liquid and PC as the electrohydrodynamic medium.

### Lamination by Ionic Liquid-Polymer Composite

**FIGURE 3:**
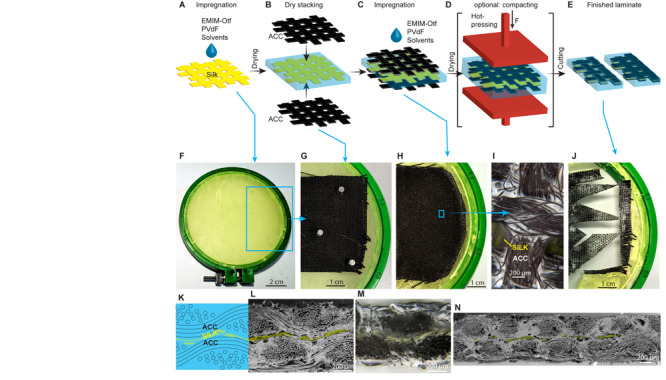
Fabrication of the ACC-silk-ACC laminate. **(A)** Impregnation of woven silk fabric. **(B)** Dry-stacking of textile layers. **(C)** Impregnation of the textiles for consolidation of the laminate. **(D)** Hot-pressing for compaction of the laminate. **(E)** Cutting the laminate to shape. **(F–H)** Photos of impregnated silk, stacked textiles, and impregnated laminate, respectively. **(I)** Close-up photograph of the ACC electrode impregnated with porous polymeric composite. **(J)** A patterned laminate, demonstrating the continuous fibrous network across a textile. The cross-section structure of the laminate is recalled in schematic **(K)** and referenced to a close-up scanning electron micrograph in **(L)** and optical micrograph in **(M)**. The long-range ordering of the laminate layers is given in scanning electron micrograph in **(N)**. The silk fibers are false-colored in yellow in **(L,N)**.

First, silk fabric was tautened on a frame to facilitate better handling. The silk was impregnated by applying the casting solution using a paintbrush (Figures 3A,F). The silk was then exposed to a stream of warm air until the decrease in reflectivity indicated the evaporation of the solvents. The impregnated fabric was then checked for pinholes by looking against the light; the impregnation step was repeated until no pinholes were found.

Next, two same-size electrodes were cut out of ACC (FM10 L100, 120 g m^–2^, 900–1100 m^2^ g^–1^, thickness 0.5 mm, Long Life for Art), and aligned on both sides of the impregnated silk (Figures 3B,G). Optionally, the three layers were fixed with polyethylene-coated magnets, as in Figure 3G. Then, the casting solution heated to 70°C was cast on the ACC electrode surface and spread out using a ceramic spatula. The impregnation process was repeated on both sides of the laminate until the ACC is fully contained within the polymeric composite (Figures 3C,H). As a result, a polymeric network continuous throughout the laminate formed. The optical micrograph of the laminate surface Figure 3I confirmed the formation of a polymeric composite in-between the fibers of ACC. It was not necessary to dose a precise amount of casting solution, as the next step removed any excess.

The laminate was then compacted using a hot plate press (Figure 3D). The laminate was checked in-between the pressing stages for thickness variations using a thickness caliper – the plateaued thickness and a smooth surface indicated a fully compacted laminate. The temperature and pressure were gradually increased up to 180°C and 18.5 bar, respectively. 5 × 20 mm samples were cut after each pressing step using a sharp scalpel. The laminate was finally cut to the desired shape (Figures 3E,J). Figure 3K recalls the laminate structure, referenced to a zoomed-in SEM and optical micrographs in Figures 3L,M, respectively. SEM microghraph in Figure 3N shows a typical compacted laminate cross-section. The silk fibers, false-colored as yellow in Figures 3L,N, are located in the middle of the laminate. The woven ACC electrodes conformed to each other, forcing the relatively thinner silk layer to conform to the ACCs separation line.

### Lamination by Carbon-Ionic Liquid-Polymer Composite

A variant of the laminate was prepared identical to described above, except a suspension of granular carbon was used for ACC impregnation. The suspension was prepared as follows. First, we dissolved 2 g of poly(vinylidene fluoride-co-hexafluoropropylene) (PVdF-HFP, Sigma-Aldrich) in 24 ml of 4-methyl-2-pentanone (MP, Alfa Aesar) (part A). Then, 2 g of carbon black (BP2000, Cabot) [20–50 nm particle size (Weingarth et al., 2014)] and 2 g of EMIM-Otf in 10 ml of MP was mixed (part B). Then, parts A and B were mixed, and 10 ml of MP was added. The suspension was homogenized using an ultrasonic probe (UP200S, Hielscher Ultrasound Technology) at a 50% duty cycle and 60% power for 30 min. Finally, the suspension was stirred overnight on a hotplate at 70°C.

### Characterization

The scanning electron micrographs were obtained using a Hitachi TM3000 microscope equipped with a back-scattered electron detector at an acceleration voltage of 15 kV. The cross-section samples were obtained by first cooling down the samples by immersing them into boiling liquid nitrogen and then sliced using a cooled scalpel blade. The silk fibers were false-colored for visual guidance.

The optical micrographs were acquired using a Sony A6300 camera with a 10 × objective; the field-of-depth was enhanced using the focus-stacking technique in Picolay software.

The electrochemical impedance of the laminate was measured using a BioLogic BP-300 potentiostat/galvanostat/FRA in two-electrode mode.

Actuation was measured by generating voltage waveforms using National Instruments’ PCI-6036E DAQ device and LabVIEW programming environment. The output current was amplified using an OPA548T operational amplifier in the voltage-follower configuration. The actuation was registered using a Keyence LK-G82/LK-G3001P laser displacement meter.

Spring-loaded clamps with contacts made of gold were used for actuation and impedance measurement.

The actuator material was characterized at room temperature (≈22°C) and relative humidity about 50%.

### Impedance Fitting

The impedance spectra were fitted using BioLogic EC-Lab Z-fit software. Randomize (30k cycles) + Levenberg-Marquardt fitting (up to 300k cycles) were used. In the case of limited convergence, the low- and high-frequency parts of the spectra were fitted piecewise.

## Results and Discussion

**FIGURE 4:**
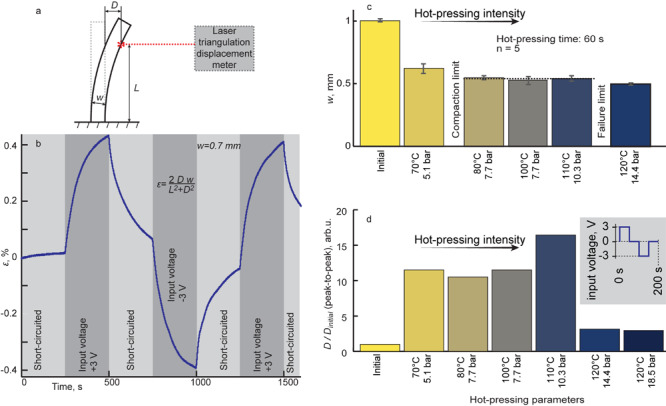
Actuation characteristics. **(a)** Bending actuator characterization set-up. **(b)** The typical transient strain of the non-hot-pressed laminate in response to 1-mHz modified square wave voltage at 3-V amplitude. **(c)** Laminate thickness evolution upon exposure to an increasing hot-pressing intensity, showing the compaction and failure limits. **(d)** Actuation increase upon increasing hot-pressing intensity.

The actuation of the compacted ACC-silk-ACC laminate with thickness *w* was measured in bending configuration, as shown in Figure 4a. For reasons of reference to similar materials, the deflection *D* at the distance *L* from the contacting clamps was converted to strain ε according to the formula *ε* = 2*Dw*/(*L*^2^ + *D*^2^) (Sugino et al., 2009). In this work, the laminate was constructed out of the thinnest commercially available stock of textile electrodes and reinforcement. The thickness of a single layer of ACC and silk textiles read 0.4–0.5 and 0.03 mm, respectively, measured by clamping the textiles in-between flat plates. A non-pressed ACC-silk-ACC laminate measured 0.7–1.0 mm in thickness (*w*), corresponding to an approximate sum of the thicknesses of its components. Naturally, a large volume proportion of polymeric composite or void filled with air remains in-between the threads; thus, a significant reduction in thickness by compaction is expected upon hot-pressing. An actuator of large thickness needs to develop a proportionally larger ε for achieving the same displacement as thinner laminates would. Even before hot-pressing, the ACC-silk-ACC laminate (*w* = 0.7 mm) developed 0.83% peak-to-peak strain in response to 1 mHz modified square-wave voltage of 3 V peak amplitude, as shown in Figure 4b. This *ε* value is well-comparable to other ionic electroactive actuators with activated carbon electrodes (Kosidlo et al., 2013; Kaasik et al., 2017). However, as the key difference, the swelling of the relatively sparsely located and undulating carbon fibers of ACC, as shown in Figures 3I,N, is unlikely to contribute significantly to total actuation. Instead, the actuation effect is predominately attributed to liquid flow within the polymeric composite in-between the carbon fibers. The surface image of the laminate in Figure 3I demonstrates favorable placement of the polymeric composite in-between carbon fibers and fiber bundles. Notably, the polymeric composite in Figure 3I appears white and opaque, indicating the abundance of empty pores that scatter light near the surface.

Open porosity in the polymer facilitates liquid mobility within the polymeric network. The polymeric network contained an active fluid, EMIM-Otf, deposited from the casting solution. The EMIM-Otf – PVdF ratio in the casting solution resulted in a significant ratio of empty pores after the evaporation of the solvents. Partially filled pores are a prerequisite for electrohydrodynamically controlled swelling gradients. The electrode exposed to an incoming liquid flux swelled the polymeric composite surrounding the ACC. The mass conservation implied displacement of liquid from the opposite electrode, where the polymeric network was subjected to capillary contraction.

The performance of an electrohydrodynamically swollen actuator is directly linked to the length and tortuosity of the ion-transport pathway between the opposing electrodes. The electrolyte ions travel toward the surfaces of the oppositely polarized electrodes, to be immobilized at the surface, as recalled in the principle of capacitive charging in Figure 2. The actuator’s geometry, as seen in Figure 3K, suggests a large spread in the electrode-to-electrode distance: a few threads of silk separate the closest instances (∼0.05 mm), whereas the ions have to travel more than 0.5 mm from one surface to another. This is in contrast to alternative electrohydrodynamic pumps, actuators, or robot construction strategies (Cacucciolo et al., 2017). Nevertheless, extremely short ion-channels have been used to construct extremely-low-voltage (0.5 V) electrohydrodynamic pumps (Shin et al., 2011), yet with Faradaic, metallic electrodes, not well-matched mechanically for actuators in bending configuration. As a practical message, the actuation in the given configuration is primarily limited by the electrochemical stability potential window of the electrolyte, ∼3 V in the case of EMIM-Otf. Additionally, this potential window is close to the maximum input voltage to the laminate, as the membrane thickness between the nearest parts electrodes is as narrow as not allow building a significant field within.

Given the fixed set of ACC electrode and silk separating reinforcement, the maximum compaction thickness was investigated. At maximum compaction, the polymeric composite is expected to fill the initially occurring voids in-between the carbon fibers best and thus yield the highest performance in the strain. Figure 4c shows the laminate thickness at increasing hot-pressing intensity. Both temperature and pressure were varied. It is demonstrated that pressing at 80°C and 7.7 bar for 60 s yielded a laminate with *w* = 0.54 ± 0.02 mm, not changing up to 110°C and 10.3 bar, thus indicating to maximum compaction thickness of the laminate. Indeed, Figure 3L shows that a laminate with minimum gaps was achieved. Exceeding 110°C and 10.3 bar did introduce further compaction, but likely with the cost of loss in electronic insulation between the electrodes.

The SEM micrograph of a hot-pressed laminate (110°C, 10.3 bar) shown in Figure 3L revealed a mostly compacted laminate with only a few voids. A more compact laminate is expected to have a positive effect on actuation for two reasons. First, the laminate thickness *w* decreases, yielding a more significant deflection at an identical strain value. Second, as the working principle is based on liquid transfer along with the porous structure of the polymeric composite, a uniform distribution of the polymeric composite increases the number of effective pores, in turn resulting in faster actuation at any input voltage. Indeed, Figure 4d reveals an increasing trend in actuation magnitude, referenced to the actuation of the initial, uncompacted laminate. Hot-pressing at 100°C and 7.7 bar yielded the highest deflection, whereas the actuation capability abruptly decreased upon more intensive pressing conditions, indicating to short-circuiting of the electrodes as the most probable failure mode.

**FIGURE 5:**
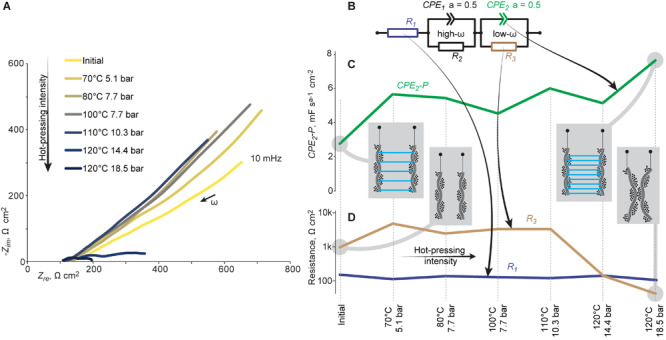
Impedance evolution upon hot-pressing. **(A)** Impedance spectra of the ACC-silk-ACC laminate exposed to hot-pressing at an increased intensity; ω denotes angular frequency. **(B)** Equivalent circuit of the laminate used for fitting. **(C,D)** *CPE_2_-P*, *R*_1_, and *R*_3_ plotted against the hot-pressing intensity. Insets in **(C,D)** illustrate the proposed mechanisms.

The effect of hot-pressing on the electrical parameters of the ACC-silk-ACC laminate was investigated using impedance spectroscopy. Indeed, Figure 5A indicates a drastic change in impedance between the ACC electrodes. Notably, although the ACC electrodes were considered capacitive, the low-frequency end of the spectra, 10 mHz, did not reach to the frequency range limited by the capacitance of the electrodes, indicated by the slope deviating from -90 degrees toward zero by up to a few tens of degrees only. Instead, the slope roughly -45 degrees was observed, indicating to ion-transfer between the electrodes as the limiting factor. Consequently, the equivalent circuit shown in Figure 5B was proposed to describe the laminate (still at a frequency above 10 mHz). The equivalent circuit consists of three elements in series. First, the resistor *R*_1_ represents energy dissipation in the ACC electrode and in the bulk of electrolyte at a frequency as high as not causing the ions to become mobile. Next, two blocks of constant-phase elements (CPEs), each in parallel with a resistive element, are to follow. The high-frequency block of *CPE*_1_ —— *R*_2_ is attributed to ion mobility within the porous network in ACC electrodes, whereas *CPE*_2_ —— *R*_3_ represents ion mobility within the porous polymeric network in-between the ACC electrodes. The exponents of both CPEs were fixed to 0.5, corresponding to mass-transfer-controlled processes.

The proportional component of *CPE*_2_, *CPE_2_-P*, shown in Figure 5C, showed a more than two-fold increase upon an increased hot-pressing intensity, suggesting to represent an increase in the number of effective porous ion-transport pathways between the electrodes. Notably, the *CPE*_2_ proportional component increased even when the short-circuiting was observed at the most intensive hot-pressing condition. Indeed, *R*_3_, the element parallel to *CPE*_2_ and attributed to short-circuiting, decreased drastically above 110°C and 10.3 bar (Figure 5D), consistent with the thickness and actuation decrease in Figures 4C,D, respectively. The parameter *R*_1_ (Figure 5D) showed only a minor decline, indicating that the failure mechanism was not a loss of contact at intensive hot-pressing.

**FIGURE 6:**
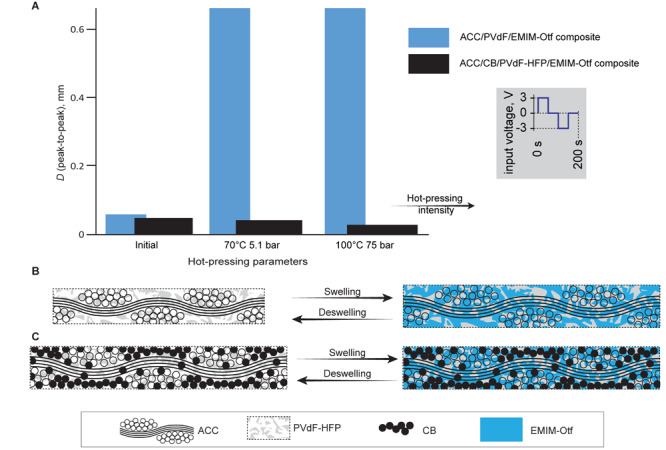
**(A)** The effect of carbon powder introduced in the polymeric composite within ACC electrodes on actuation in response to 5-mHz modified square wave voltage at 3-V amplitude. **(B)** A large amount of porous polymer in-between ACC fibers results in a high strain, whereas in **(C)**, a higher load of the active carbon particles decreases strain due to high carbon-carbon particle interaction.

To further look into the electrodes’ contribution to actuation, we investigated a laminate with its electrodes constructed of ACC filled with a suspension of hi-SSA non-monolithic carbon black (CB) powder. Unexpectedly, Figure 6A reveals the performance-decreasing effect of CB introduced into the voids in ACC electrodes. An additional carbon loading is expected to increase the areal capacitance at the cost of the porous polymeric network volume. An increase in actuation magnitude would indicate the significant contribution by carbon electroswelling (Kaasik et al., 2013), whereas a decrease in actuation supports the hypothesis of swelling of the polymeric binder being primarily responsible.

Figure 6B explains the favorable case of a large volume of polymeric binder situated in-between monolithic, fibrous carbons, promising a significant strain. In the case of the polymeric matrix loaded with CB particles, as in Figure 6C, continuous carbon-to-carbon force chains are formed. Although the increased carbon load does attract and store ions to its surface, the strong inter-particle interaction between CB particles as well as between CB particles and ACC prevents exertion of swelling effects in the polymeric network in-between the carbon particles (also acting as a binder for the CB particles) for a pronounced actuation.

We specifically do not want to claim CB (or other non-monolithic carbons) being detrimental to actuation *per se*; the results in Figure 6 should be seen as specific to the current configuration that also incorporated monolithic fibers. According to our interpretation, also given in Figures 6B,C, the addition of CB decreased the relative amount of swelling polymer material per area and prevented the polymer from exerting a significant pressure for a composite volume change. In other words, the polymer experienced higher stiffness of the surrounding carbon matrices due to percolation and developed higher internal stress due to swelling, without expression via bending.

## Conclusion

Electrically controlled electrolyte and solvent exchange (i.e., mass transfer) between the sides of a sub-millimeter-thick laminated textile provides a flexible platform for a variety of wearable robotic applications, including shape morphing. The textile platform is strategic for three reasons. First, the textile provided mechanical (silk) and electrical (ACC) integrity within the laminate, facilitating easy fabrication. Second, micrometer-scale-diameter fibers with a meter-scale length dimension enable straightforward integration of actuators into wearable appliances. Third, abundant biomaterial-derived textiles support a high level of chemical safety in touch with humans.

Commercially available conductive and insulating woven textiles were used as electrodes and reinforcements, respectively. Three-textile-layer electroactive laminates of 0.7–1.0 mm initial thickness were compacted up to 0.54 mm by hot-pressing, increasing the displacement. In the textile-based laminate, the expression of volumetric effects in woven ACC electrodes was suppressed; instead, the actuation effect is attributed to reversible swelling of the polymeric composite. The ionic liquid is electrohydraulically displaced in the thickness direction of the laminate, increasing swelling on the side with incoming liquid flux, whereas decreasing on the opposite. The measured maximum strain difference, 0.8%, is comparable to previous configurations of bending ionic actuators, further suggesting a high contribution by reversible polymer swelling to actuation.

## Data Availability Statement

All datasets generated for this study are included in the article/supplementary material.

## Author Contributions

IU contributed to the design of experiments and performed the majority of experiments. FK contributed to the design of experiments and revised the manuscript. UJ performed SEM observations, took part in the discussions, and revised the manuscript. AA took part in the discussions and revised the manuscript. IM developed the concept, contributed to the design of experiments, and wrote the manuscript.

## Conflict of Interest

The authors declare that the research was conducted in the absence of any commercial or financial relationships that could be construed as a potential conflict of interest.
